# Role of Calbindin-D28k in Diabetes-Associated Advanced Glycation End-Products-Induced Renal Proximal Tubule Cell Injury

**DOI:** 10.3390/cells8070660

**Published:** 2019-06-30

**Authors:** Kuo-How Huang, Siao-Syun Guan, Wei-Han Lin, Cheng-Tien Wu, Meei-Ling Sheu, Chih-Kang Chiang, Shing-Hwa Liu

**Affiliations:** 1 Department of Urology, College of Medicine and Hospital, National Taiwan University, Taipei 100, Taiwan; 2 Institute of Nuclear Energy Research, Atomic Energy Council, Taoyuan 325, Taiwan; 3 Institute of Toxicology, College of Medicine, National Taiwan University, Taipei 100, Taiwan; 4 Division of General Surgery, Department of Surgery, Shuang Ho Hospital, Taipei Medical University, New Taipei City 235, Taiwan; 5 Institute of Biomedical Sciences, National Chung Hsing University, Taichung 402, Taiwan; 6 Departments of Integrated Diagnostics & Therapeutics and Internal Medicine, College of Medicine and Hospital, National Taiwan University, Taipei 100, Taiwan; 7 Department of Medical Research, China Medical University Hospital, China Medical University, Taichung 404, Taiwan; 8 Department of Pediatrics, National Taiwan University Hospital, Taipei 100, Taiwan

**Keywords:** advanced glycation end products, renal proximal tubule, calbindin-D28k, renal fibrosis, diabetic nephropathy

## Abstract

Diabetes-associated advanced glycation end-products (AGEs) can increase extracellular matrix (ECM) expression and induce renal fibrosis. Calbindin-D28k, which plays a role in calcium reabsorption in renal distal convoluted tubules, is increased in a diabetic kidney. The role of calbindin-D28k in diabetic nephropathy still remains unclear. Here, calbindin-D28k protein expression was unexpectedly induced in the renal tubules of *db*/*db* diabetic mice. AGEs induced the calbindin-D28k expression in human renal proximal tubule cells (HK2), but not in mesangial cells. AGEs induced the expression of fibrotic molecules, ECM proteins, epithelial-mesenchymal transition (EMT) markers, and endoplasmic reticulum (ER) stress-related molecules in HK2 cells, which could be inhibited by a receptor for AGE (RAGE) neutralizing antibody. Calbindin-D28k knockdown by siRNA transfection reduced the cell viability and obviously enhanced the protein expressions of fibrotic factors, EMT markers, and ER stress-related molecules in AGEs-treated HK2 cells. Chemical chaperone 4-Phenylbutyric acid counteracted the AGEs-induced ER stress and ECM and EMT markers expressions. Calbindin-D28k siRNA in vivo delivery could enhance renal fibrosis in *db*/*db* diabetic mice. These findings suggest that inducible calbindin-D28k protects against AGEs/RAGE axis-induced ER stress-activated ECM induction and cell injury in renal proximal tubule cells.

## 1. Introduction

Diabetes is a metabolic disease resulting from β-cell destruction/insulin deficiency (type 1 diabetes) or insulin resistance/insulin secretory defect (type 2 diabetes) or other specific diabetic types, causing hyperglycemia [1]. Under a long-term condition of hyperglycemia, diabetes may cause several complications or diseases such as coronary heart disease, cerebrovascular disease, retinopathy, neuropathy, and nephropathy [1,2]. Under these complications, the diabetic nephropathy is wildly noted because of the higher prevalence in diabetic patients and the increased risk of death [3]. High-level blood glucose, free fatty acid and advanced glycation end-products (AGEs) are generally considered as the risk factors for diabetic patients. The AGEs products are mainly formed through a non-enzymatic Maillard reaction between reducing sugars and amine residues on proteins, lipids, or nucleic acids. The accumulation of AGEs has been found to elicit the progressive alterations in renal architecture including the metalloproteinases changes, enhancement of extracellular matrix proteins formation or interaction with plasma protein, which accelerate the dysfunction of kidneys in diabetic patients [4]. AGEs also induce the disruption of calcium homeostasis and the induction of oxidative stress and inflammatory responses that ultimately lead to renal injury or fibrosis [4,5,6]. Although the correlation between physiological changes and AGEs levels are defined, the molecular mechanisms of AGEs-related renal proximal tubule cell injury remain to be clarified.

Calbindin-D28k, which possesses four high affinity calcium binding sites and has a high-concentration in the distal tubule of kidney and in the brain and pancreas, plays the roles in the trans-cellular calcium transportation and the modulation of intracellular calcium concentrations of regulating renal calcium absorption and maintaining renal function [7]. The expression of calbindin-D28k has also been shown to be significantly changed in the kidneys of streptozotocin-induced diabetic rats [8] and OVE26 diabetic mice [9]. Another study showed that calbindin-D28k protected against cyclosporine A-induced renal proximal tubular cell cytotoxicity through its buffering effects on the regulation of intracellular calcium concentrations [10]. Rabinovitch et al. have also found that calbindin-D28k protects against cytokine-mediated β-cell death via the inhibition of free radical formation [11]. These findings suggested that calbindin-D28k might play the roles in the improvement of disorders in several target organs, including the kidney. However, the role of calbindin-D28k in diabetic nephropathy still remains unclear. We further hypothesized that the inducible calbindin-D28k might be involved in the process of AGEs-induced renal proximal tubule cell injury and fibrosis. In this study, therefore, we investigated the role of calbindin-D28k in AGEs-induced renal proximal tubule cell injury using cell and animal models.

## 2. Materials and Methods

### 2.1. Animals

Twelve-week-old male C57BL/6J *db*/*db* (BKS.Cg- Dock7^m^ +/+ Lepr^db^/J; diabetic littermate) and control *db*/*m*+ male mice were used. The *db*/*db* and control *db*/*m*+ mice were obtained from Jackson Laboratory (Bar Harbor, ME, USA). The *db*/*db* mice, which the blood glucose level was over than 300 mg/dL, were used in the experiments. The mice were housed in the controlled conditions (22 ± 2 °C and 40–60% relative humidity with a cycle of 12 h light/12 h dark) with free access to food and water. The animal experiments were approved by the Animal Research Committee of College of Medicine, National Taiwan University and followed the regulations of Taiwan and National Institutes of Health (NIH, USA) guidelines for the care and welfare of laboratory animals. Animals were humanely treated and with regard for alleviation of suffering. Animals were anesthetized by inhalational application of a mixture gas of isoflurane (3%) (Baxter Healthcare of Puerto Rico, Guayama, PR, USA) and oxygen (97%), and then euthanized.

### 2.2. Immunohistochemistry

The 4-μm-thick paraffin-embedded renal tissue sections were used. The antigen retrieval sections were blocked by 5% bovine serum albumin at room temperature for 1 h and incubated with the primary antibodies for AGEs (1:500; abcam, Cambridge, MA, USA) and calbindin-D28k (1:500; Cell Signaling Technology, Danvers, MA, USA). In some experiments, the renal tissue sections were stained with Masson’s trichrome stain for renal fibrosis [6].

### 2.3. Double Immunofluorescence Staining

The 4-μm-thick renal tissue sections were undergone the deparaffinization and rehydration procedure. The sections were retrieved by an autoclave in citrate buffer (pH 6.0) for 45 min. The sections were rinsed in PBST (115 mM NaCl, 3.6 mM KCl, 1.3 mM KH_2_PO_4_, 25 mM NaHCO_3_, and 0.05% tween 20; pH 7.4), and then incubated with primary antibodies for calbindin-D28k (Cell Signaling Technology) and AQP-1 (abcam) overnight. Finally, the sections were stained by the anti-rabbit fluorescein isothiocyanate (FITC) or anti-mouse tetramethylrhodamine (TRITC) fluorescent secondary antibodies (Sigma-Aldrich, St. Louis, MO, USA) for 1 h. The counterstain was performed by using Hoechst 33,258 (Sigma-Aldrich).

### 2.4. Cell Culture

Human kidney proximal tubular cell line (HK2), mouse kidney mesangial cell line (MMC; MES-13), and Madin-Darby canine kidney distal tubular cells (MDCK) were obtained from American Type Culture Collection (Manassas, VA, USA). HK-2 cells were maintained in Dulbecco’s modified Eagle’s medium (DMEM; GIBCO, Grand Island, NY, USA)/Ham’s F-12 Nutrient Mixture medium (F12; GIBCO) at a ratio of 1:1. MMC and MDCK cells were maintained in DMEM. The fresh medium was supplemented with 10% fetal bovine serum (FBS, GIBCO) and antibiotics (100 IU/mL penicillin, 100 μg/mL streptomycin, and 0.25 μg/mL amphotericin B). Cells were cultured at 37 °C and 5% carbon dioxide (CO_2_).

### 2.5. Preparation of AGEs

AGEs were prepared and purified from the incubation of bovine serum albumin (BSA) and D-glucose as described previously [12] with a modification. Bovine serum albumin (BSA, 100 mg/mL) and D-glucose (0.5 M) were incubated in phosphate buffer (0.2 M, pH7.4) at 37 °C. After reaction for 8 weeks under a sterile condition, the mixture solution was collected. The unincorporated glucose was then removed by dialyzing membrane against phosphate-buffered for 2 times during 24 h. Finally, the AGEs were passed through the 0.22 μm filter to remove the contaminants. An Ultraflex-III MALDI-TOF/TOF mass spectrometer (Bruker, Billerica, MA, USA) was used to identify the AGEs. The concentration of AGEs was determined by a BCA protein assay kit (Thermo Fisher Scientific, Waltham, MA, USA).

### 2.6. Protein Extraction

Cells were washed by the phosphate-buffered saline (PBS; pH 7.4) and harvested by a cold radioimmunoprecipitation (RIPA) buffer (20 mM Tris-base, 150 mM NaCl, 1 mM EDTA, 1 mM EGTA and 1% NP40; pH 7.4) with the protease/phosphatase inhibitor cocktail. Cell proteins were isolated at 13,000 rpm, 4 °C, for 30 min. The protein concentration was determined by a Bicinchoninic acid (BCA) protein assay kit (Thermo Fisher Scientific).

### 2.7. Western Blot Analysis

The proteins of cells or renal tissues were detected in 10% or 12% sodium dodecyl sulfate polyacrylamide gel electrophoresis (SDS-PAGE) and were transferred onto the polyvinylidene difluoride membrane (0.22 μm). The membranes were washed with Tris-buffered saline Tween-20 (TBST) buffer followed by blocking with 5% milk for 1 h, and then incubated with the primary antibodies for AGEs (1:1000; abcam, Cambridge, MA, USA), connective tissue growth factor (1:1000; connective tissue growth factor (CTGF), fibronectin, receptor for AGE (RAGE), β-actin (1:1000; Santa Cruz Biotechnology, Santa Cruz, CA, USA), calbindin-D28k (CaBP-D28K), vimentin, CHOP, GRP78, snail, IRE1, PERK, phosphorylated eIF2α, eIF2α (1:1000; Cell Signaling Technology, Beverly, MA, USA), E-cadherin (BD Biosciences, San Jose, CA, USA), and α-smooth muscle actin (1:1000; α-SMA, Sigma-Aldrich, St. Louis, MO, USA) overnight at 4 °C. The membranes were then incubated in anti-rabbit or anti-mouse secondary antibodies (1:5000; Santa Cruz Biotechnology) conjugated with horseradish peroxidase for 1 h and detected by enhanced chemiluminescence (BioRad, Hercules, CA, USA). Densitometric analysis was assessed using Image J software, version 1.52a (National Institutes of Health, Bethesda, MD, USA).

### 2.8. RNA Interference

A short interfering RNA (siRNA) for calbindin-D28k [Stealth siRNAs (Set of 3) HSS101302, HSS101303, HSS101304] and a negative control siRNA (Stealth siRNA Negative Control Kit, Catalog number: 12935100) were purchased from Invitrogen (Invitrogen, Carlsbad, CA, USA). Cells were seeded on 6-well plates and were transfected with scramble or calbindin-D28k siRNA by using Lipofectamine RNAiMAX transfection reagent (Thermo Fisher Scientific). The siRNA/Lipofectamine medium was removed after 6 h of incubation. Cells were then cultured for 48 h at 37 °C. The siRNA sequences targeting calbindin-D28k were as follows: sense 1: 5′-ACU GAC CAC AGU GGC UUC AUA GAA A-3′; antisense 1: 5′-UUUCUAUGAAGCCACUGUGGUCAGU-3′; sense 2: 5′-ACUGAAGGAUCUGUGCGAGAAGAAU-3′; antisense 2: 5′-AUUCUUCUCGCACAG AUCCUUCAGU-3′; sense 3: 5′-GAAGAACAUAAUGGCUUUGUCGGAU-3′; antisense 3: 5′-AUCCGACAAAGCCAUUAUGUUCUUC-3′.

### 2.9. Delivering siRNA in Vivo

The optimized modification and endotoxin free of CaBP-D28K or scrambled siRNAs (Invitrogen) were dissolved in RNase/DNase free water. Male 8-week-old *db*/*db* mice were randomized into two groups (scramble and siRNA). The siRNA-treated mice received 530 μL of kidney In Vivo Transfection reagent (Altogen Biosystems) in combination with 90 μg CaBP-D28K siRNA. Control animals received 530 μL of In Vivo Transfection reagent in combination with 90 μg scramble siRNA. Diabetic mice were rapidly intraperitoneal (i.p.) injected scramble or CaBP-D28K siRNA, once per 3 days for a total of 6 times. All mice were sacrificed 48 h after last injection. The kidneys were collected for Western blot, Masson’s trichrome staining, and immunohistochemistry assay. For quantification of Masson’s trichrome staining, the collagen levels were analyzed in a blinded fashion with digital image analysis software (ImageJ version 1.52a, National Institutes of Health).

### 2.10. Statistical Analysis

Data are presented as means ± SEM. The difference between control and treatment groups was assessed by one-way analysis of variance (ANOVA) and followed to the Dunnett’s test. A *p*-value of less than 0.05 is considered to be a significant difference. Statistical analysis was performed by using GraphPad Prism software, version 6.0 (San Diego, CA, USA).

## 3. Results

### 3.1. Induction of Calbindin-D28k and AGEs Accumulation in the Kidney of Diabetic Mice

**Figure 1:**
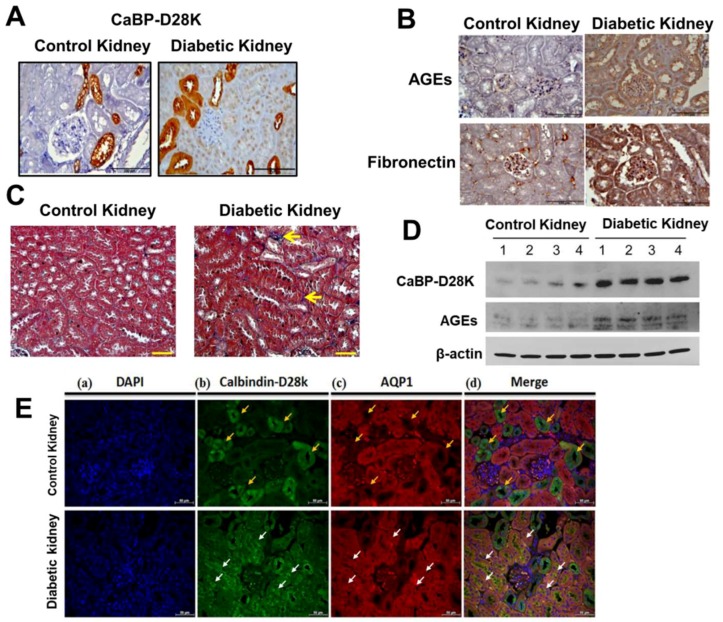
Expressions of calbindin-D28k and AGEs in the kidneys of *db*/*db* diabetic mice. The immunohistochemical stainings for calbindin-D28k (**A**) and AGEs and fibronectin (**B**) were performed in the sections of kidneys from control *db*/*m*+ mice or *db*/*db* diabetic mice. (**C**) Masson’s Trichrome staining detected the renal fibrosis in the kidneys of control and diabetic mice. Scale bar: 100 μm. (**D**) Western blotting analyzed the protein expressions of calbindin-D28k and AGEs in the kidneys of control and diabetic mice. (**E**) Expression of calbindin-D28k in the renal proximal tubules of *db*/*db* diabetic mice. The immunofluorescence double stainings for identifying nuclei (DAPI, blue; a), calbindin-D28k (green; b), and AQP-1 (proximal tubule marker) (red; c) were performed in the sections of kidneys from control *db*/*m*+ mice or *db*/*db* diabetic mice. The merge of staining was shown in d. The yellow arrows indicated for the calbindin D28k positive/AQP-1 negative regions. The white arrows indicated for the calbindin D28k positive/AQP-1 positive regions. Scale bar: 100 μm.

Previous study has indicated that 80% calbindin-D28k proteins are majorly expressed in the distal convoluted tubule cells in the rat and human kidneys [13]. The expression of calbindin-28k on diabetic renal tubules was unclear. We first investigated the induction of calbindin-D28k and AGEs accumulation in the kidney of *db*/*db* diabetic mice compared to control *db*/*m*+ mice. The data were presented in Figure 1. The immunohistochemical staining of calbindin-D28k in the kidney of *db*/*db* mice was higher than that in control mice (Figure 1A). The calbindin-D28k-positive staining was majorly observed in renal tubules, but not in the glomeruli of *db*/*db* and control mice. Moreover, both AGEs and fibronectin stainings were obviously augmented in the renal glomerulus and tubule areas of *db*/*db* mice (Figure 1B). Masson’s trichrome staining showed the interstitial fibrosis in the kidney of *db*/*db* mice (Figure 1C). Moreover, both calbindin-D28k and AGEs protein expressions were obviously augmented in the kidney of *db*/*db* mice (Figure 1D). Unexpectedly, we found that calbindin-D28k could be induced in the proximal tubules of diabetic mice. As shown in Figure 1E, calbindin-D28k-positive staining co-localized with the AQP-1 (proximal tubular marker) staining in the renal proximal tubular regions of *db*/*db* diabetic mice, but not control mice. These results suggest that calbindin-D28k expression can be enhanced in the renal proximal tubules of *db*/*db* diabetic mice.

### 3.2. AGEs Induce Calbindin-D28k Expression in Renal Proximal Tubular Cells

**Figure 2:**
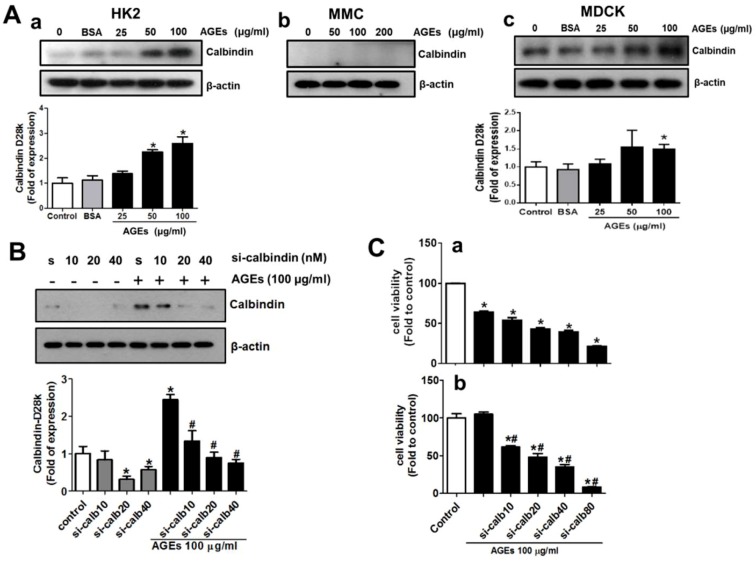
Effects of AGEs on protein expressions of calbindin-D28K in renal proximal tubule cells, mesangial cell, and distal tubule cells and calbindin-D28k silencing reduced cell viability in HK2 cells. (**A**) AGEs (25-200 μg/mL) or BSA (100 μg/mL) were added to renal proximal tubule cells (HK-2; a), mesangial cells (MMC; b), and distal tubule cells (MDCK; c) for 48 h. The protein expression was determined by Western blotting. Protein levels were quantified by densitometry and normalized by β-actin levels. Data are presented as means ± SEM (n = 5). * *P* < 0.05, vs. control group. (**B**) and (**C**) Cells were transfected with scramble (s or control; 40 or 80 nM) or calbindin-D28k siRNA (si-calbindin or si-calb5-80; 5–80 nM) for 6 h. Transfected cells were treated with or without AGEs (100 μg/mL) for 48 h. Protein expression of calbindin-D28k was determined by Western blotting (**B**). Protein levels were quantified by densitometry and normalized by β-actin levels. Cell viability was determined by MTT assay (**C**). Data are presented as means ± SEM (n = 5). * *P* < 0.05, vs. scramble control group. # *P* < 0.05, vs. AGEs+scramble group.

We next tested whether AGEs treatment triggered the induction of calbindin-D28k expression in the human renal proximal tubule cells (HK-2). The data were presented in Figure 2A. AGEs (25-100 μg/mL) treatment for 48 h significantly up-regulated calbindin-D28k protein expression in HK2 cells in a dose-dependent manner (Figure 2A-a). The constitutive calbindin-D28k protein expressions in both HK2 cells and mesangial cells (MES-13) without any treatment were very low (Figure 2A-a,A-b). However, AGEs treatment did not induce the calbindin-D28k protein expression in the mesangial cells (Figure 2A-b). In contrast, the constitutive calbindin-D28k protein expression in MDCK distal tubular cells without any treatment was high (Figure 2A-c). AGEs at the concentration of 100 μg/mL significantly increased the calbindin-D28k protein expression in MDCK cells (Figure 2A-c).

### 3.3. Interference of Calbindin-D28k Decreases Cell Viability and Enhances AGEs-Induced Fibrotic Responses

**Figure 3:**
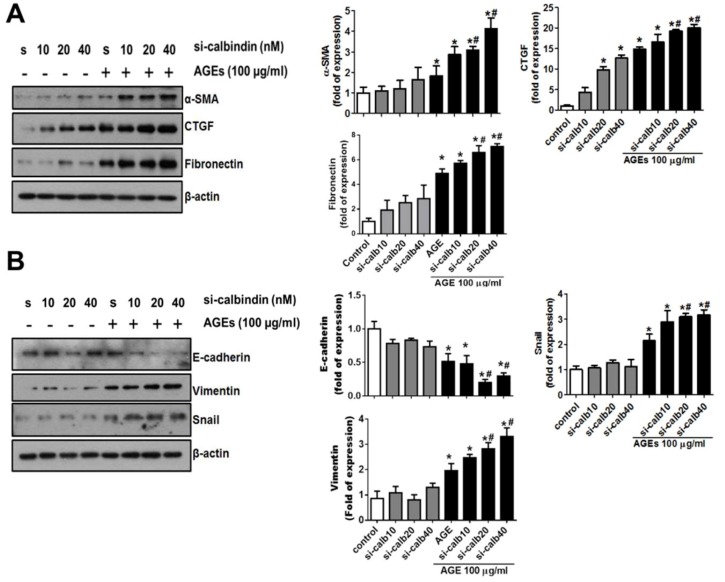
Calbindin-D28k silencing enhanced AGEs-induced fibrotic signals in HK2 cells. Cells were transfected with scramble (s or control; 40 nM) or calbindin-D28k siRNA (si-calbindin or si-calb10-40; 10–40 nM) for 6 h. Transfected cells were treated with or without AGEs (100 μg/mL) for 48 h. (**A**) Protein expressions of α-SMA, CTGF, and fibronectin were determined by Western blotting. (**B**) Protein expressions of E-cadherin, vimentin, and Snail were determined by Western blotting. Protein levels were quantified by densitometry and normalized by β-actin levels. Data are presented as means ± SEM (n = 5). * *P* < 0.05, vs. scramble control group. # *P* < 0.05, vs. AGEs+scramble group.

To investigate the roles of the inducible calbindin-D28k in renal proximal tubule cells after AGEs treatment, the expression of calbindin-D28k was interfered by using siRNA transfection. The data were presented in Figure 2B. The results showed that the transfection of calbindin-D28k siRNA markedly decreased the AGEs-induced calbindin-D28k protein expression in a dose-dependent manner (Figure 2B). HK2 cell viability was significantly and dose-dependently reduced by calbindin-D28k siRNA transfection with or without AGEs treatment (Figure 2C). AGEs at the concentration of 100 μg/mL did not affect the cell viability in HK2 cells under normal culture condition. Moreover, the accumulation of AGEs has been shown to increase extracellular matrix and fibrosis [5]. We next tested the role of calbindin-D28k in the fibrotic signals in AGEs-treated proximal tubule cells. The data were presented in Figure 3. Calbindin-D28k silencing could significantly enhance the AGEs-induced fibrotic protein expressions (α-SMA, CTGF, and fibronectin) (Figure 3A) and activation of epithelial-mesenchymal transition (EMT) (decreased E-cadherin and increased snail and vimentin) (Figure 3B). These results suggest that calbindin-D28k knockdown promotes AGEs-induced EMT induction and renal fibrosis in renal proximal tubule cells.

### 3.4. Role of RAGE in the AGEs-Induced Expressions of Calbindin-D28k and Fibrotic Markers in HK2 Cells

**Figure 4:**
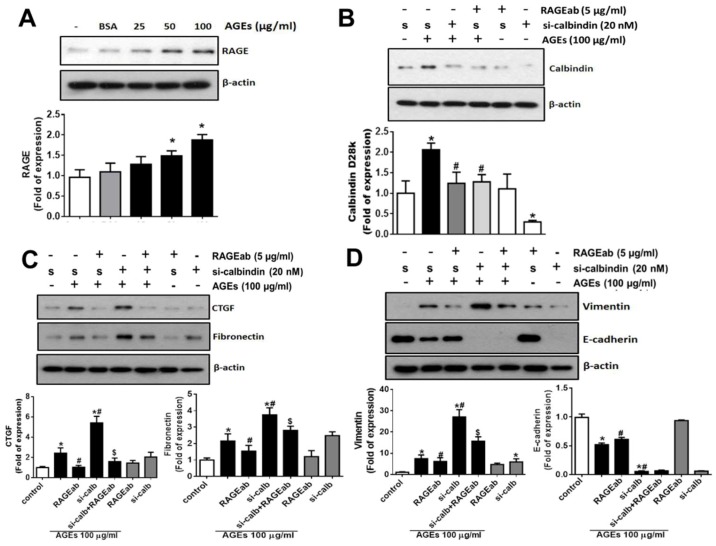
RAGE blockade suppressed AGEs-induced fibrotic signals and calbindin-D28k in the presence or absence of calbindin-D28k silencing in HK2 cells. (**A**) Cells were treated with AGEs (25–100 μg/mL) or BSA (100 μg/mL) for 48 h. Protein expressions of RAGE in HK-2 cells were determined by Western blotting. In (**B**–**D**), cells were transfected with scramble (s or control; 40 nM) or calbindin-D28k siRNA (si-calbindin or si-calb; 40 nM) for 6 h. Transfected cells were treated with RAGE neutralizing antibody (RAGEab, 5 μg/mL) for 1 h, and then treated with AGEs (100 μg/mL) for 48 h. Protein expressions of calbindin-D28k (**B**), CTGF and fibronectin (**C**), and vimentin and E-cadherin (**D**) in HK-2 cells were determined by Western blotting. Protein levels were quantified by densitometry and normalized by β-actin levels. Data are presented as means ± SEM (n = 4). * *P* < 0.05, vs. control or scramble control group. # *P* < 0.05, vs. AGEs+scramble group. $ *P* < 0.05, vs. AGEs+calbindin-D28k siRNA group.

AGE-RAGE signal pathway is known to play an important role in the pathogenesis of diabetic nephropathy [14]. Therefore, we next investigated the role of AGE-RAGE axis in calbindin-D28k induction and fibrogenesis-related proteins expression, the neutralizing antibody for RAGE, which caused the blockade of RAGE-ligand interaction [15], was used. The data were presented in Figure 4. AGEs treatment up-regulated the protein expression of RAGE in HK-2 cells (Figure 4A). The inductions of calbindin-D28k (Figure 4B) and fibrotic markers (CTGF and fibronectin) (Figure 4C) and the changes of EMT markers (Figure 4D) induced by AGEs in HK2 cells could be significantly inhibited by treatment with RAGE neutralizing antibody. The RAGE neutralizing antibody could also inhibit the calbindin-D28k-siRNA-enhanced CTGF, fibronectin (Figure 4C), and vimentin (Figure 4D) protein expressions, although it did not affect the decreased E-cadherin expression (Figure 4D) in AGEs-treated HK2 cells. These results indicate that AGE-RAGE axis is involved in the calbindin-D28k induction and fibrogenesis-related proteins expressions in renal proximal tubule cells.

### 3.5. Calbindin-D28k Deficiency Enhances AGEs-Induced ER Stress Signaling in HK2 Cells

**Figure 5:**
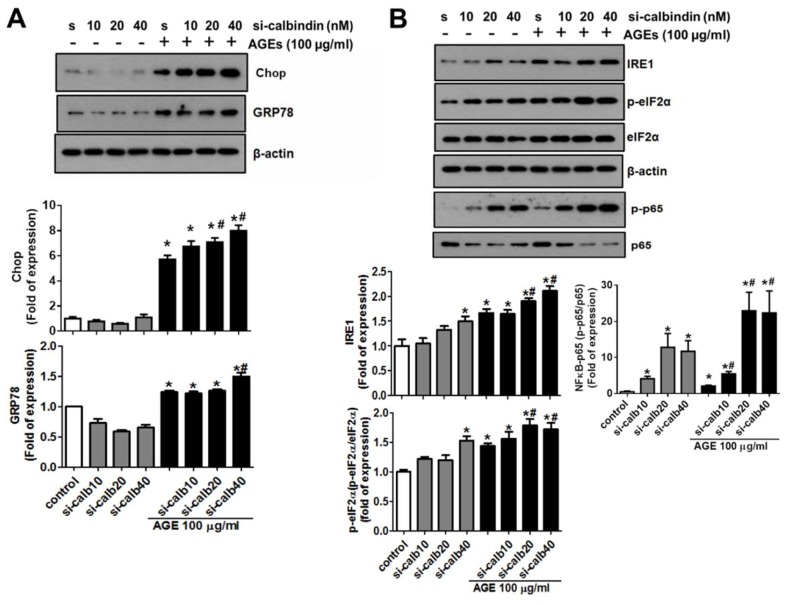
Calbindin-D28k silencing enhanced AGEs-induced ER stress signals in HK2 cells. Cells were transfected with scramble (s or control; 40 nM) or calbindin-D28k siRNA (si-calbindin or si-calb10-40; 10–40 nM) for 6 h. Transfected cells were treated with or without AGEs (100 μg/mL) for 48 h. Protein expressions of CHOP and GRP78 (**A**) and IRE1, phosphorylated eIF2α (p-eIF2α), eIF2α, NFκB-p65, and phosphorylated NFκB-p65 (**B**) were performed by Western blotting. Protein levels were quantified by densitometry and normalized by β-actin or eIF2α or NFκB-p65 levels. Data are presented as means ± SEM (n = 5). * *P* < 0.05, vs. scramble control group. # *P* < 0.05, vs. AGEs + scramble group.

A previous study indicated that ER stress-related molecules were involved in the AGEs (10–160 μg/mL)-induced glomerular mesangial injury [12]. The role of calbindin-D28k in AGEs-induced ER stress in proximal tubule cells is unclear. We next investigated whether calbindin-D28k regulated the activation of AGEs-induced ER stress signaling in HK2 cells. The data were presented in Figure 5. Calbindin-D28k siRNA transfection significantly enhanced AGEs-induced protein expressions of CHOP, GRP78, IRE1, and phosphorylated-eIF2α (Figure 5A,B). We also found that phosphorylation of NFκB-p65 could also be enhanced by calbindin-D28k siRNA in AGEs-treated HK2 cells (Figure 5B). These results suggest that calbindin-D28k silencing enhances AGEs-induced cells ER stress in HK cells.

### 3.6. 4-Phenylbutyric Acid (4-PBA) Counteracts the Effects of Calbindin-D28k Deficiency on ER Stress and EMT Signals in HK2 Cells

**Figure 6:**
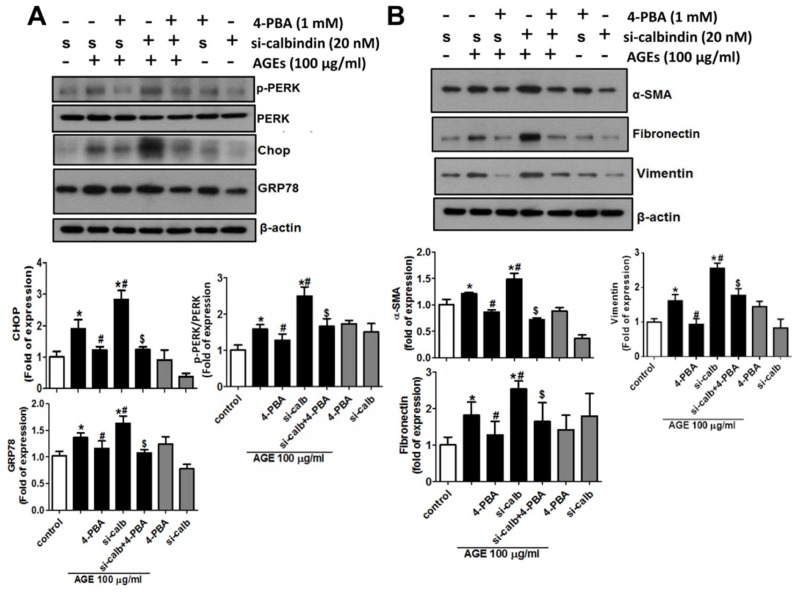
4-PBA counteracted AGEs-induced and calbindin-D28k silencing-enhanced ER stress and fibrotic signals in HK2 cells. Cells were transfected with scramble (s or control; 40 nM) or calbindin-D28k siRNA (si-calbindin or si-calb; 40 nM) for 6 h. Transfected cells were treated with 4-PBA (1 mM) for 1 h, and then treated with AGEs (100 μg/mL) for 48 h. Protein expressions of CHOP, GRP78 and PERK (**A**) and α-SMA and fibronectin and vimentin (**B**) in HK-2 cells were determined by Western blotting. Protein levels were quantified by densitometry and normalized by β-actin levels. Data are presented as means ± SEM (n = 5). * *P* < 0.05, vs. control or scramble control group. # *P* < 0.05, vs. AGEs + scramble group. $ *P* < 0.05, vs. AGEs + calbindin-D28k siRNA group.

Next, a chemical chaperone, 4-PBA, was used to investigate the effects of calbindin-D28k on AGEs-induced ER stress and fibrotic signals in HK2 cells. The data were presented in Figure 6. Calbindin-D28k siRNA transfection significantly enhanced AGEs-induced protein expressions of CHOP, GRP78, and PERK in HK2 cells, which could be significantly reversed by 4-PBA (Figure 6A). Moreover, the administration of 4PBA significantly counteracted AGEs-induced protein expressions of α-SMA, fibronectin, and vimentin in HK2 cells (Figure 6B). These results indicate that the enhancement of calbindin-D28k silencing on ER stress PERK/eIF2α/CHOP axis and EMT signals in AGEs-treated HK2 cells can be antagonized by chemical chaperone 4-PBA.

### 3.7. Calbindin-D28k siRNA in Vivo Delivery Enhanced Renal Fibrosis in db/db Diabetic Mice

**Figure 7:**
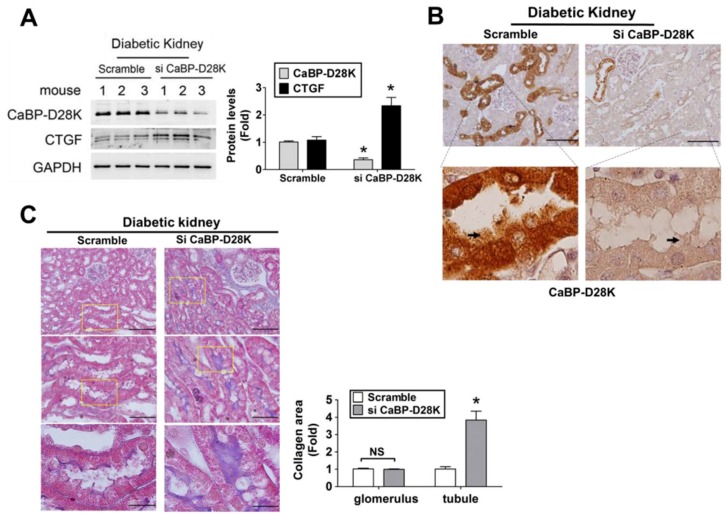
Calbindin-D28k siRNA in vivo injection increased renal fibrosis in the kidneys of *db*/*db* diabetic mice. (**A**) The protein expressions of calbindin-D28k and CTGF in the kidneys of control (scramble) diabetic mice and siRNA delivery diabetic mice were determined by Western blotting. Three representative mice per group were shown. Protein levels of immunoblotting were quantified by densitometry and normalized by GAPDH levels. Data were presented as means ± SEM (n = 6). * *P* < 0.05, siRNA versus control (scramble). (**B**) Immunohistochemical staining for calbindin-D28k in the kidneys of *db*/*db* diabetic mice without or with in vivo siRNA delivery. In lower panel, the arrow indicated the brush border of proximal tubule. (**C**) Masson’s Trichrome staining detected the renal fibrosis in si-calbindin-D28k and scramble injected diabetic mice. A panoramic view is displayed on the top panel and a magnification of the indicated region is displayed on the middle and bottom panel. Upper panel: scale bar: 100 μm; Middle panel: scale bar: 50 μm; Lower panel: scale bar: 20 μm. The semi-quantitative assessment of collagen levels with five random areas per section was determined by ImageJ software. Data are presented as means ± SEM (n ≥ 3). * *P* < 0.05, siRNA versus scramble control.

To confirm the results of in vitro experiments, calbindin-D28k siRNA in vivo delivery was determined in *db*/*db* diabetic mice. The data were presented in Figure 7. Calbindin-D28k protein expression (Figure 7A) and immunohistochemical staining (Figure 7B) were markedly decreased, but the CTGF protein expression (Figure 7A) was significantly elevated in the kidneys of *db*/*db* mice with siRNA in vivo delivery as compared to scramble control. We also tried to magnify the scale to observe the proximal tubules in kidney sections. We found that calbindin-D28k staining was clearly shown in proximal tubules in which the brush borders in some proximal tubule cells were shed (Figure 7B). Moreover, Masson’s trichrome staining showed that renal fibrosis in tubular area was markedly enhanced in *db*/*db* mice with calbindin-D28k siRNA delivery (Figure 7C). The Masson’s trichrome staining was also clearly shown in proximal tubules of kidney sections at higher magnification (Figure 7C, lower panel).

## 4. Discussion

In *db*/*db* diabetic mice, we have unexpectedly found that AGEs and calbindin-D28k protein expression could be induced in the proximal renal tubules. Calbindin-D28k is known to be high-concentration in the renal distal tubules, but not in the proximal tubules. We further found that AGEs markedly induced the calbindin-D28k expression in the proximal tubule cells. Therefore, we have interest in studying this phenomenon. We investigated the role of calbindin-D28k in AGEs-induced renal proximal tubule cell injury using models of human renal proximal tubule cells and *db*/*db* diabetic mice.

Diabetic nephropathy is one of the critical complications of diabetic mellitus. Persistent hyperglycemia condition may accelerate the development of the end-stage renal disease (ESRD). The 20–40% of diabetic patients has been estimated to develop a chronic kidney disease that for a significant number of patients can lead to ESRD [16]. Studies indicated that the accumulation of AGEs in diabetic patients with renal disease is two folds higher than diabetic patients without renal disease [17]. AGEs-RAGE axis has been suggested to play an important role in the development of diabetic nephropathy [17,18]. The beneficial effects of AGE lowering or RAGE inhibitors against diabetic nephropathy have been demonstrated in experimental models [18]. In this study, we found that AGEs significantly increased the protein expressions of ECM proteins, fibrotic molecules, ER stress-related molecules, and EMT-related molecules in human renal proximal tubular HK-2 cells, which could be significantly reversed by RAGE neutralizing antibody. These results indicate that AGEs-RAGE axis may play an important role in early diabetic nephropathy and cause proximal tubule cell injury and EMT induction.

Interesting, the expression of calbindin-D28k, a high-affinity cytosolic calcium binding protein, has been shown to be increased in the kidney and it was associated with the increase in the fractional excretion for calcium in the distal convoluted tubule of streptozotocin-induced diabetic rats [19]. Calbindin-D28k protein has also been found to be broadly and markedly expressed in the kidneys of OVE26 diabetic mice [9]. The alteration in calbindin-D28k has been reported to cause the disturbance of calcium balance in the cerebellar Purkinje cells of spinocerebellar ataxia type 1 (SCA1) transgenic mice [20] and in the hippocampal CA1 region and dentate gyrus granule cell layer of streptozotocin-induced diabetic rats [21]. Moreover, 17β-estradiol treatment could protect dopaminergic neurons via the increase of calbindin-D28K expression in a Parkinson’s disease mouse model [22]. These findings suggested that calbindin-D28k plays a protective role in the neurons. In the presented study, we demonstrated that AGEs dose-dependently induced the protein expression of calbindin-D28k in HK2 cells, but not in mesangial cells. Silencing of calbindin-D28k by siRNA enhanced the expressions of ER stress-related molecules and fibrotic molecules (α-SMA, CTGF, and fibronectin) and EMT induction in AGEs-treated HK2 cells. These results suggested that calbindin-D28k not only constitutively expresses in the distal convoluted tubules and collecting ducts, but also can be induced by AGEs stimulation in the proximal tubules. We also found that the protein expressions of AGEs and calbindin-D28k were significantly and markedly induced in the kidneys of *db*/*db* diabetic mice. Unexpectedly, calbindin-D28k could be induced in the proximal tubules of the *db*/*db* diabetic mouse kidney, but not control mouse kidney. We further used calbindin-D28k siRNA to silence calbindin-D28k expression in the kidneys of *db*/*db* diabetic mice and found that CTGF protein expression and renal fibrosis were markedly enhanced. These in vivo results are consistent with the in vitro findings.

Previous studies indicated that ER stress activation is an important feature for the process of recovery, adaptation, and apoptosis during diabetic nephropathy. Several ER stress-associated signals, including CHOP and/or IRE1-mediated JNK pathway, can cause renal cells apoptotic death, prolonged oxidative stress-induced kidney damage and/or kidney fibrosis [23,24,25]. CHOP is a major downstream molecule of the PERK-eIF2α-ATF4 signal pathway and the ATF6-related unfolded protein responses (UPR) pathway. CHOP deficiency has been reported to prevent inflammatory responses, renal cell apoptosis, and renal fibrosis in a chronic kidney disease (CKD) rat model [26,27]. On the other hand, the activation of IRE1, a branch of UPR, has also been found to be involved in the inflammatory responses via pathways including JNK and NF-κB signaling cascades [28]. The loss of cytosolic Ca^2+^ balance might cause ER stress and activate UPR including CHOP and IRE-1-associated signals, leading to the cell death or rebuild the normal ER function [29]. The alteration in the expressions of CHOP and IRE-1 has also been demonstrated to disturb the calcium homeostasis in several pathological conditions, including diabetes [30,31].

A previous study has indicated that ER stress inducer can induce EMT in human peritoneal mesothelial cells [32]. ER stress signaling cascade has also been shown to promote the ECM remodeling and fibrosis induction via the activation of pro-apoptotic pathways the induction of inflammatory responses [33], or disruption of calcium homeostasis [7]. In contrast, a recent study found that the ER stress inducer, thapsigargin, treatment markedly decreased, but tunicamycin increased the expression of calbindin D28k in the kidney cells (Madin-Darby bovine kidney cells, MBDK cells) [34]. In the present study, our data revealed that calbindin-D28k gene silencing with siRNA enhanced the expressions of ER stress-related molecules, including CHOP, GRP78, IRE1, p-PERK, and p-eIF2α, in AGEs-treated renal proximal tubule cells and also enhanced the expression of fibrotic molecules. These results suggested AGEs treatment disrupted the calcium homeostasis and then aroused the changes of ER stress signaling in proximal tubular cells. Furthermore, 4-PBA, a chemical chaperone, is known to prevent ER stress-related inflammatory responses, suppressing the progression of diabetic nephropathy [35]. We further found that 4-PBA effectively prevented the AGEs-induced signaling molecules for ER stress, EMT, and fibrosis in the absence and presence of calbindin-D28k siRNA transfection. These results suggest that calbindin-D28k plays a protective role in calcium homeostasis against AGEs-induced ER stress in renal proximal tubule cells.

Shin et al. have indicated that ER stress can serve as a target for ameliorating EMT via Smad2/3-related signals in human peritoneal mesothelial cells [32]. The activation of Snail signaling has also been shown to be involved in the development of renal fibrosis [36]. Cyclosporine has been found to be capable of activating a TGF-β-independent Snail expression via JNK signaling in the human renal epithelial cells [37]. AGEs/RAGE axis is capable of activating TGF-β1-independent Smad2/3 signaling through an ERK/p38/MAPK pathway to cause renal fibrosis and inflammation [38]. The role of calbindin-D28k in AGEs/RAGE axis-induced ER stress-activated molecular signals of Smads and MAPKs in renal proximal tubule cells needs to be further clarified in the future.

The previous studies of reference [39] and our research team [40] have indicated that the accumulation of ECM proteins is the main cause of diabetic glomerular fibrosis in *db*/*db* mice. We have also demonstrated that exposure of AGEs significantly induces cell apoptosis via an ER stress-triggered signaling pathway in mesengial cells (MES-13) [12]. We have also found that exposure of AGEs can induce the expression of CTGF, a fibrosis-induced factor, in mesengial cells. Overexpression of calbindin-D28k has been reported to provide cytoprotective effects in several cell models including neuronal cells [41,42] and pancreatic beta cells [43]. Calbindin-D28k is known to be high-concentration in the distal tubule of kidney and in the brain and pancreas [7]. It has been shown that the sites of calbindin-D28k distribution in the normal rat or human kidneys are the distal convoluted tubules, connecting tubules, and collecting ducts [44,45]. Karan et al. have also found that in the normal kidney of *Martes foina*, the immunohistochemical positive cells for calbindin-D28k were located in the distal tubules, but not in the glomeruli, proximal tubules, and loops of Henle [46]. In the present study, the constitutive calbindin-D28k protein expressions in both proximal tubular HK2 cells and glomerular mesangial cells (MES-13) under normal condition (without any treatment) were very low. Our results are consistent with the previous findings. On the other hand, it has been demonstrated that the immunohistochemical positive cells for calbindin-D28k were significantly enhanced in the distal convoluted tubules, collecting ducts, and proximal convoluted tubules in the kidney of OVE26 diabetic mice (a transgenic model of Type 1 diabetes) [9]. In the present study, we also found that calbindin-D28k can be enhanced in the renal tubules of *db*/*db* diabetic mice (a model of Type 2 diabetes). The calbindin-D28k was not located in the diabetic glomeruli. Therefore, we further hypothesized that the inducible calbindin-D28k might be involved in the process of AGEs-induced renal proximal tubule cell injury and fibrosis. The results suggest that inducible calbindin-D28k protects against AGEs/RAGE axis-induced ER stress-activated ECM induction and cell injury in renal proximal tubule cells.

Calbindin D28k possesses four high calcium binding sites and consecutively expressed with higher level in the distal renal tubular region to maintain the calcium homeostasis [7]. Chiappisi et al. have recently shown that both thapsigargin and tunicamycin, which are the ER stress inducers, significantly decrease and increase the mRNA expression of calbindin (CALB1), respectively, in the renal distal tubular cells (Madin-Darby bovine kidney cells), whereas the mRNA expression of ATPase plasma membrane Ca^2+^ transporting 1 (PMCA1) is not changed or slightly decreased [34]. They suggested that the induction of ER stress presumably did not alter the plasma membrane Ca^2+^ transport in renal distal tubular cells; ER stress-evoked ER Ca^2+^ depletion-disturbed intracellular Ca^2+^ homeostasis may be involved in the alteration of CALB1 expression. In the present study, we unexpectedly found that AGEs induced calbindin-D28k expression in renal proximal tubular cells (HK2 cells) and calbindin-D28k silencing enhanced the ER stress signaling and the expression of fibrosis-related molecules. However, the role of calbindin-D28k in the regulation of ER Ca^2+^ levels in renal proximal tubules under AGEs or diabetic condition is still unclear and needs further investigation.

AGEs with various concentrations have been shown to affect the cell growth and function in various cell types. AGEs (AGE-BSA) at 100 μg/mL have been found to reduce the viable cell numbers and induced apoptotic cell death at 250 μg/mL in retinal pericytes [47]. It has been shown that AGEs (AGE-BSA, 25–200 μg/mL) can induce apoptosis in pancreatic islet endothelial cells [48]. AGEs [AGE-human serum albumin (HSA)] at 100 μg/mL have been found to induce apoptosis in some cancer cell lines [49]. Chiang et al. (2016) have found that 40–160 μg/mL AGEs (AGE-BSA) significantly reduced mesangial cell viability and induced mesangial cell apoptosis [12]. In this study, we found that AGEs (AGE-BSA) at the concentrations of 25–100 μg/mL induced the calbindin-D28k expression in HK2. AGEs induced the expression of fibrotic molecules, ECM proteins, epithelial-mesenchymal transition (EMT) markers, and endoplasmic reticulum (ER) stress-related molecules in HK2 cells. The concentrations of AGEs used in this study are reasonable.

Moreover, the loss of proximal tubule brush border has been observed in the kidneys of streptozotocin/nicotinamide-induced type 2 diabetic mouse model [50] and *db*/*db* diabetic mouse model [51]. Therefore, in the present study, the brush borders in proximal tubules of kidney sections may shed during diabetic nephropathy, which stained tubule lacks the brush borders (Figure 1).

## 5. Conclusions

The results of this study showed that the inducible calbindin-D28k plays an adaptive and protective role against AGEs-induced ER stress and the renal fibrosis signals that occurs afterwards in the proximal tubule cells. Calbindin-D28k can be induced in the renal proximal tubules in *db*/*db* diabetic mice. Calbindin-D28k may serve as a therapeutic strategy for the improvement of diabetic nephropathy. However, more evidence from animal studies is needed to confirm the role of calbindin-D28k in diabetic nephropathy in the future. Moreover, further investigation also needs to clarify the role of calbindin-D28k in calcium homeostasis of renal tubules during diabetes.
